# The role of epigenetic modifications in Colorectal Cancer Metastasis

**DOI:** 10.1007/s10585-022-10163-w

**Published:** 2022-04-16

**Authors:** Riya Su, Xinlin Wu, Liang Tao, Changshan Wang

**Affiliations:** 1 Department of pharmacology, Zhongshan School of Medicine, Sun Yat-sen University, Guangzhou, China; 2 Department of General Surgery, the Affiliated Hospital of Inner Mongolia Medical University, Huhhot, China; 3 The State Key Laboratory of Reproductive Regulation and Breeding of Grassland Livestock, School of Life Sciences, Inner Mongolia University, Hohhot, China

**Keywords:** Epigenetics, DNA methylation, Histone modification, RNA methylation, CRC metastasis

## Abstract

Distant metastasis is the major contributor to the high mortality rate of colorectal cancer (CRC). To overcome the poor prognosis caused by distant metastasis, the mechanisms of CRC metastasis should be further explored. Epigenetic events are the main mediators of gene regulation and further affect tumor progression. Recent studies have found that some epigenetic enzymes are often dysregulated or mutated in multiple tumor types, which prompted us to study the roles of these enzymes in CRC metastasis. In this review, we summarized the alteration of enzymes related to various modifications, including histone modification, nonhistone modification, DNA methylation, and RNA methylation, and their epigenetic mechanisms during the progression of CRC metastasis. Existing data suggest that targeting epigenetic enzymes is a promising strategy for the treatment of CRC metastasis.

## Introduction

Colorectal cancer (CRC) is the world’s fourth most deadly cancer, and the disease-specific mortality rate is nearly 33% in the developed world[1, 2]. According to statistics provided by the National Cancer Center of China, it accounted for approximately 319,486 new cancer cases and 164,959 cancer-related deaths in males and 235,991 new cases and 121,203 deaths in females in 2020[3]. Many risk factors contribute to CRC, including increasing age, male sex, previous colonic polyps or previous CRC, unhealthy lifestyle or eating habits, inflammatory bowel disease and hereditary syndromes such as Lynch syndrome and familial adenomatous polyposis[1]. Although the outcomes of CRC patients have been improved with advanced technologies and therapies, the estimated death rate of CRC patients is still increasing year by year[3, 4].

The major contributor to the high mortality rate of CRC is distant metastasis. Approximately 25% of patients present with metastases at initial diagnosis, and almost 50% of patients with CRC ultimately develop metastases[5], which drove us to assess the mechanism contributing to CRC metastasis to prevent it from occurring. Tumor metastasis is a multistep process that presents as an invasion-metastasis cascade, including local invasion, intravasation and survival in the circulation, arrest at distant organs and extravasation into the parenchyma of distant tissues, formation of micrometastases and restart of the proliferative program[6]. Several biological processes contribute to CRC metastasis. Epithelial-to-mesenchymal transition (EMT), a major metastatic contributor, is a biological process in which epithelial cells transform into cells with a mesenchymal phenotype through specific procedures. EMT increases the metastatic potential of carcinoma cells by increasing their migratory and invasive capacities, which facilitates their movement out of primary tumor sites and into the circulation[7]. Cancer stem cells (CSCs), a small subset of cells with self-renewal and tumor-initiating ability within tumors, have also been proven to play an important role in CRC metastasis. Compared to non-CSCs, CSCs exhibit enhanced metastasis-related traits, such as motility, invasiveness and resistance to apoptosis[8]. Researchers found that colon cancer stem cells (CCSCs) are critical for the formation and maintenance of liver metastasis[9]. However, there is also a different view that CCSCs are indispensable for the outgrowth, but not the establishment, of metastases[10]. In addition to the above processes, the roles of the activation of multiple signaling pathways, metabolic reprogramming and immune escape in CRC metastasis cannot be ignored.

Wisniewski and coworkers compared the proteomes of formalin-fixed, paraffin-embedded (FFPE) samples containing colonic mucosa, primary colon tumors and nodal metastatic compartments. Although dramatic changes in the proteome were noted in the primary tumors compared to their matched normal tissues, the differences between the tumors and the metastases were much subtler[11]. Dynamic epigenetic modifications may be able to account for the mechanisms of CRC metastasis that cannot be explained simply by proteomic changes. At present, studies related to epigenetic modifications in the context of metastasis mainly focus on the following aspects: (a) epigenetic modification of EMT-inducing transcription factors (EMT-TFs), such as Snail and Slug, and their downstream effectors; (b) epigenetic modification of components of key CSC pathways that can also regulate the EMT process, such as the Wnt/β-catenin, Hedgehog, and Notch signaling pathways. (c) epigenetic modification of key effectors or metabolic enzymes in metabolic pathways and epigenetic modification of molecules that are crucial to recruit immunosuppressive cells. A large number of studies have shown that epigenetic enzymes are often dysregulated or mutated in cancer, and the subsequent changes in epigenetic modifications can further change gene transcription and protein stability to promote metastasis[12, 13]. In this review, we summarized several major epigenetic enzymes and their modifications and discussed their molecular mechanisms and clinical applications in the process of CRC metastasis.

## Posttranslational modification in CRC metastasis

Posttranslational modifications include histone modifications and nonhistone modifications, which play a pivotal role in the development and progression of CRC. According to the different functional groups added, posttranslational modifications can be classified as acetylation, methylation, phosphorylation, ubiquitination, sumoylation, succinylation, ADP ribosylation, lactylation, isonicotinylation and so forth. In the next part of this review, more attention will be given to enzymes that mediate methylation and acetylation and their roles in CRC metastasis (Table 1). Lysine and arginine are the most common modification sites, and these modifications are catalyzed by acetylases and methylases. Simply, lysine and arginine residues on the N-terminal tails of histones are subject to methylation and acetylation, and these alternations further regulate the chromatin structure, thus affecting gene transcription. For nonhistone proteins, acetylation and methylation may regulate protein stability and further affect protein activity and function.

## Acetylation and Deacetylation

### CREB-binding protein (CBP)/p300

CBP and its homolog p300 are two acetyltransferase enzymes in humans and most higher eukaryotes[14]. CPB/p300 are multifunctional proteins that can acetylate diverse signaling effectors, enhancer-associated regulators and all four core histones[15]. Approximately 21,000 acetylation sites on 5,300 proteins can be acetylated by CBP/p300, and CBP/p300-regulated sites are significantly enriched for transcription and chromatin regulation[15].

To date, several studies have reported that CBP/p300 participates in the mechanism of CRC metastasis. LncRNA SATB2-AS1 recruits p300 and accelerates the p300-mediated acetylation of H3K27 and H3K9 at the SATB2 promoter to upregulate SATB2, which inhibits the invasion and migration of CRC cells[16]. CREPT, a tumor-related gene significantly associates with the poor overall survival (OS) of CRC, promotes the CRC metastasis by recruiting p300 and enhancing the acetylation of H3K27 and H4 in the c-myc promoter region to directly control its transcriptional efficacy[17, 18]. In addition to promoting histone acetylation, CBP/p300 also promotes the acetylation of nonhistone proteins to participate in the process of CRC metastasis. CREPT, as described above, can also enhance the interaction between p300 and β-catenin to promote p300-mediated acetylation and stability of β-catenin[18]. DOT1L is a new substrate of CBP, whose acetylation at K358 protects DOT1L from degradation to promote CRC metastasis[19]. ArhGAP30, a Rho GTPase-activating protein, promotes CRC cell migration in a p53-dependent manner, which upregulates p53 activity by enhancing the acetylation of p53 at the Lys382 site in the presence of p300[20]. A recent study found that CRC tumor-initiating cells (TICs) expressing CD110, a thrombopoietin (TPO)-responsive homodimeric receptor, mediated liver metastasis[21]. TPO expression strengthens the interaction between p300 and LRP6, which accelerates acetylation of LRP6 at the K802 site to activate LRP6 and further stimulate self-renewal of CD110 + TICs, thus promoting CRC metastasis[22]. Although these results indicate that CBP/p300 has an essential role in the mechanism of CRC metastasis, the relationship between CBP/p300 and some clinical features, such as tumor-node-metastasis (TNM) stage, survival rate and metastasis rate, is inconsistent[23–25]. These results indicate that CBP/p300 prefers to act as a downstream effector to assist the function of some important proteins rather than as a metastasis-initiating protein.

### P300/CBP associated factor (PCAF)

PCAF, an acetyltransferase of the GNAT family, was first described by its competition with adenoviral oncoprotein E1A for binding to CBP/p300[26]. PCAF controls gene transcription by promoting the acetylation of H3K9[27], H3K14[28], and H4[29]; moreover, PCAF accelerates the acetylation of some nonhistone proteins, such as EZH2[30], PGK1[31], p53[32], and PTEN[33], to take part in the process of tumor progression. Previously, several studies reported that CXCL12 was involved in the metastasis of CRC[34, 35]. Romain and coworkers identified that both PCAF and CXCL12 were downregulated in colorectal tumor samples and found that a PCAF expression plasmid could significantly increase the CXCL12 gene expression level, which drove them to speculate that acetylation of the CXCL12 promoter may explain this phenomenon[36]. β-Catenin also plays a key role in CRC metastasis[37]. PCAF increases the transcriptional activity and nuclear accumulation of β-catenin, and this process greatly depends on the HAT2 domain of PCAF[38]. A pull-down assay revealed that PCAF not only coimmunoprecipitated with β-catenin but also promoted the acetylation of the K19 and K49 sites of β-catenin[38]. Encouraged by this result, researchers manipulated PCAF expression in CRC cells. The results were consistent with what they expected: knockdown of PCAF promoted cell differentiation and inhibited cell migration and tumor growth in vivo[38].

### The histone deacetylase (HDAC) family

**Fig. 1:**
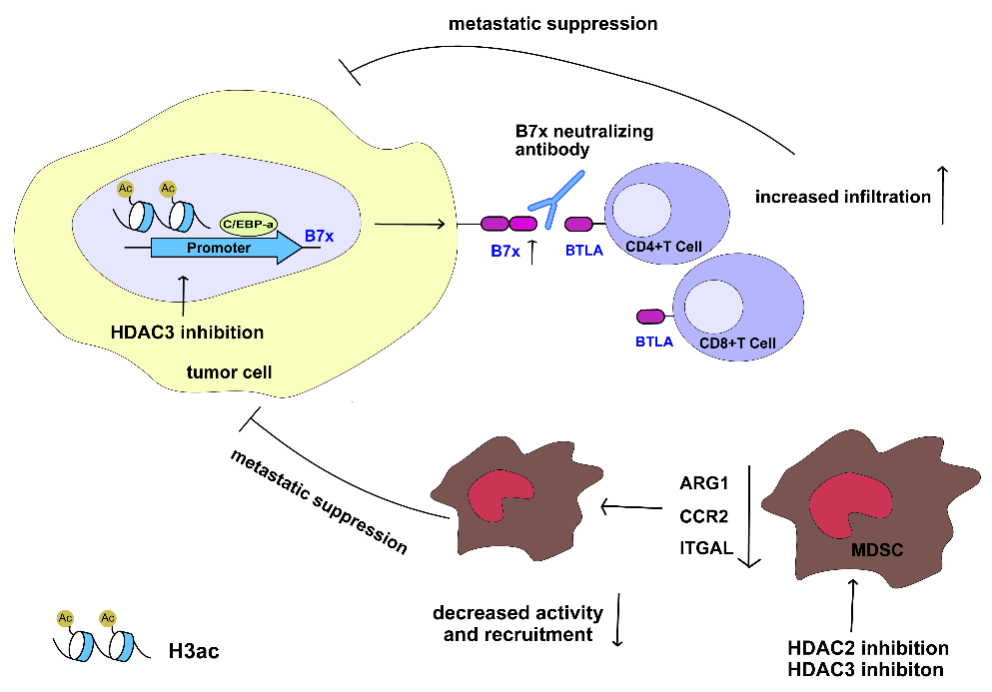
HDAC inhibition represses tumor metastasis by reprogramming the tumor immune microenvironment

HDACs are a family of proteases that can be divided into four classes. HDACs deacetylate histones and lead to chromatin condensation, which might cause decreased or increased gene transcription. Several researchers have reported the clinical significance of HDAC family in CRC. They found that the positive rates of HDAC1, HDAC2, and HDAC3 expression were 36.4%, 57.9%, and 72.9%, respectively, in CRC tissues, and elevated HDAC levels significantly correlated with reduced patient survival[39, 40]. Furthermore, they also reported that the expression of all three HDAC isoforms was higher in the tumors with distant metastasis (HDAC1, *P* = 0.037; HDAC2, *P* = 0.045; HDAC3, P = 0.062), suggesting that they can play a prometastatic role in CRC[39]. An in vivo tumor xenograft assay showed that the HDAC inhibitor JNJ-26,481,585 strongly induced pan-H3 acetylation in tumor tissues and fully inhibited the growth of C170HM2 colorectal liver metastases[41]. These results indicated the prognostic impact of these HDACs and the treatment value of HDAC inhibitors. HDACs modulate CRC metastasis through various mechanisms. MMP3 and Claudin-1, proteins mainly associated with the invasion potential of cancer cells, can be epigenetically or nonepigenetically regulated by HDACs[42, 43]. It has been reported that HDACs can also reprogram the tumor immune microenvironment to modulate the metastatic process (Fig. 1). HDAC3 inhibitor treatment increases the Ac-H3 level at B7x promoter and promotes the interaction of C/EBP-α with the B7x promoter to upregulate the expression of B7x, an immune checkpoint molecule crucial to the immune escape of tumors, which contributes to HDAC inhibitor resistance in CRC[44]. Combined treatment with an HDAC inhibitor and B7x neutralizing antibody increased the infiltration of CD8 + and CD4 + T cells in CRC tissue from metastatic tumor xenografts and reduced the lung metastasis of the CRC model[44]. Nair and coworkers compared the RNA-sequencing data between CRC tissue-derived and normal tissue-derived immature myeloid-derived suppressor cells (I-MDSCs), a class of immune suppressive cells crucial to tumor metastasis, and found that 148 of the upregulated genes in tumor-infiltrating I-MDSCs were involved in HDAC activation and that HDAC inhibitors significantly reduced MDSC function and the expression of recruitment-associated genes ARG1, CCR2, and ITGAL[45]. All these results suggested that HDAC-mediated deacetylation might be involved in the mechanism of CRC metastasis and indicated the possibility of HDACs as targets of immune environment regulators in CRC.

HDAC3 inhibition enhances B7x expression, and combined treatment with an HDAC inhibitor and a B7x neutralizing antibody increases the infiltration of CD8 + and CD4 + T cells. HDAC2 and HDAC3 inhibition can decrease the activity and recruitment of I-MDSCs in the tumor microenvironment, which further suppresses the metastatic ability of CRC cells.

### The mammalian sirtuin (SIRT) family

The SIRT family contains seven members (SIRT1–7), which are evolutionarily conserved NAD+-dependent histone deacetylases or ADP-ribosyltransferases belonging to the class III HDAC family. Members of the SIRT family localize to different sites of the cell and are involved in many cellular processes, such as DNA repair, cell cycle regulation and cell metabolism, and the protection of cells from oxidative stress[46].

Many assays performed on patient tissues have shown that the SIRT family members are differentially expressed in CRC cancer tissues compared to adjacent normal tissues. For example, SIRT1, SIRT6 and SIRT7 are overexpressed in CRC tissues, and their upregulation is significantly associated with advanced tumor-node-metastasis (TNM) stage, a poor prognosis, decreased overall survival and disease-free survival in CRC patients[47–50]. Furthermore, the overexpression of SIRT1, SIRT6 and SIRT7 is positively correlated with lymph node or liver metastases[47, 48, 50]. Many articles have confirmed the prometastatic role of SIRT1. It was reported that Fra-1 is a component of AP-1 complex that can promote tumor-associtated EMT by directly regulating EMT-TFs, and SIRT1 enhances the EMT process in a Fra-1-dependent manner[49, 51]. However, the acetylation level of Fra-1 was not discussed in this article. Because of the crucial role of CSCs in CRC metastasis, Wang et al. found that SIRT1 could decrease H3K9ac enrichment on the promoter of miR-1185-1 and thus cause chromatin remodeling to repress miR-1185-1 expression, which further upregulated the CD24 level to promote the stemness and migration of CRC cells[52]. Some noncoding RNAs have been found to be associated with SIRT1 expression and to further contribute to the metastasis process of CRC. An in vitro assay proved that SIRT1 was a direct target of microRNA-199b and that SIRT1 further affects the acetylation level of CREB to upregulate the transcriptional activity of CRE*B*[53]. In addition, SIRT6 promotes the EMT process of CRC cells in two different ways: it acts as a reader of snail and also suppresses TET1 transcription by modulating H3K9 deacetylation[50]. Controversially, many researchers have pointed out that SIRT6 and SIRT7 play a tumor inhibitory role via a variety of mechanisms, such as antagonizing the c-myc oncogene, regulating the DNA damage response, and inhibiting JAK2/STAT3 and PTEN/AKT signaling[54–57], but their roles in CRC metastasis were not discussed in these articles.

SIRT2 and SIRT4 seem to play metastasis-inhibiting roles in CRC. Recently, IDH1 has received great attention because of its effect on cancer metabolism[58]. Researchers found that IDH1 k224 acetylation tightly controls enzymatic activity through the HIF1a-SRC axis and promotes the liver metastasis of CRC and that SIRT2 can be a potential strategy for preventing CRC liver metastasis because of its role in inhibiting the acetylation and enzymatic activity of IDH[59]. Through the immunohistochemical analysis of SIRT2 protein expression in colorectal tissue specimens, they also confirmed that SIRT2 expression was significantly decreased in CRC tissues or liver metastases compared with corresponding colorectal normal tissues[59]. Similarly, SIRT4 expression was found to be negatively associated with lymph node metastasis, lymphatic invasion, and distant metastasis[60]. Consistent with its associated clinicopathological features, SIRT4 overexpression decreased the invasion and migration ability of CRC cells by suppressing miR-200c to further affect E-cadherin expression[60]. From these articles, we can easily conclude that the roles of the SIRT family in CRC metastasis are diverse. In addition to using different kinds of CRC models, integrated analysis of multiple SIRT members and assessment of the role of the same SIRT member in different stages of CRC development, such as pre- and post-metastasis, also need to be performed.

## Methylation and demethylation

### Enhancer of zeste homolog 2 (EZH2)

The EZH2 gene encodes histone lysine N-methyltransferase, a catalytic member of the PRC2 complex, which methylates H3K9 and H3K27 at the posttranslational level, leading to the transcriptional repression of target genes. EED and SUZ12, two other subunits of PRC2, are indispensable for the enzymatic activity of EZH2[61]. Additionally, it was reported that EZH2 can also methylate H3K4 in a PRC2-independent manner to play a gene activation role[62].

Many studies have demonstrated that the mRNA and protein expression of EZH2 is significantly increased in CRC tissues compared with adjacent noncancerous tissues, and that EZH2 overexpression is closely associated with reduced OS and disease-free survival (DFS)[63, 64]. In addition, the high level of EZH2 expression has been strongly linked to both regional lymph node (p < 0.001) and distant metastasis (p = 0.004) [63, 64]. Moreover, one allelic variant (rs3757441 C/C) of EZH2 is significantly associated with shorter progression-free survival (PFS) and OS in metastatic CRC patients[65]. Such findings indicated the prognostic and therapeutic value of EZH2 for advanced CRC patients.

Many studies have verified that EZH2 plays a prometastatic role in various cancer types, and that the pharmacological inhibition of EZH2 can significantly repress the migration and invasion of the cancer cells[66, 67]. Similarly, in CRC, EZH2 can act as a downstream regulator of some important signaling pathways and noncoding RNAs, thus promoting CRC metastasis. TGF-β-MTA1-SOX4 signaling drives the EMT process to promote CRC metastasis. In this signaling axis, EZH2 acts as a downstream regulator of EMT-associated factors such as E-cadherin, ZO-1, snail, and slug[68]. Activation of the Erk/Akt signaling pathway induces EZH2 overexpression to repress the transcription of ITGα2 and E-cadherin by affecting the enrichment level of H3K27me3 on their promoters[69]. In addition, EZH2 acts as a downstream regulator of lncRNAs/miRNAs to promote CRC metastasis[70–75]. For example, lncRNA MALAT1and lncRNA SNHG14 can regulate EZH2 to further affect H3K27me3 recruitment on the E-cadherin and EPHA7 promoters, respectively[71, 74]. MiR-101 inhibits the invasion and migration of CRC cells[76]. The downregulation of miR-101 enhances the stability of EZH2 by regulating O-GlcNAcylation on EZH2, which, in turn, further reduces miR-101 expression by recruiting H3K27me3 to its promoter[76]. Although many other articles have proven that EZH2 is a downstream target of many lncRNAs/miRNAs, it has not been discussed whether EZH2 exerts its prometastatic function through histone modification[70, 72, 73, 75]. Therefore, intensive studies of the mechanism are urgently needed.

### Suppressor of variegation 3–9 homolog 1 and suppressor of variegation 3–9 homolog 2 (SUV39H1 and SUV39H2)

Suv39h1 and Suv39h2 are H3K9 selective histone methyltransferases that were first isolated and characterized in mice and identified as modulating chromatin dynamics in somatic cells[77, 78]. They epigenetically modulate functional proteins to control telomere length[79], heterochromatin organization, chromosome segregation, and mitotic progression[78, 80]. In recent years, many studies have proven that SUV39H1 and SUV39H2 play an important role in various cancer types, such as CRC[81, 82], melanoma[83], breast cancer[84], cervical cancer[85] and hematologic malignancies[86, 87].

In CRC, SUV39H1 is significantly upregulated in tumor tissues compared to normal colon tissues[88]. Overexpression of wild-type SUV39H1 increased H3K9me3 levels as well as the migration and invasion abilities of CRC cells, and this effect was eliminated when the SUV39H1-C326A mutant, which lacks enzymatic activity, was introduced[89]. Although the functional genes enriched by H3K9me3 were not discussed in this article, these results indicated the potential relation of SUV39H1 with the metastasis process of CRC. Similar to SUV39H1, it was reported that high SUV39H2 expression is strongly associated with distant metastasis and TNM stage and predicts shorter OS and PFS for CRC patients[81]. SUV39H2 enhances CRC metastasis by directly binding to the SLIT1 promoter and catalyzes H3K9me3 to suppress SLIT1 expression[81]. A small molecule inhibitor of SUV39H1 has been developed, and it can suppress human colon tumor xenograft growth in vivo[88], which confirmed the therapeutic value of SUV39H1 in CRC. Therefore, the metastasis inhibition ability of this SUV39H1 inhibitor needs to be further tested.

### Protein arginine methyltransferases (PRMTs)

PRMT1 and PRMT5 are the major members of type I and type II PRMT families that catalyze asymmetric dimethylarginine (ADMA) and symmetric dimethylarginine (SDMA) deposition on proteins. They have been reported to regulate multiple cellular processes, including transcriptional activation and repression, RNA splicing, protein synthesis, DNA damage response, signal transduction and liquid–liquid phase separation[90]. A type I PRMT inhibitor (GSK3368715) has already been shown to inhibit proliferation in patient-derived DLBCL models and several cell lines that represent the majority of tumor types. A synergistic cancer cell growth inhibition effect was observed when PRMT5 was inhibited with GSK3368715, which further indicated the therapeutic value of type I and type II PRMTs[91].

In CRC, PRMT1 promotes cell migration and invasion through histone arginine methylation and nonhistone arginine methylation. PRMT1-mediated H4R3me2a directly recruits SMARCA4 to promote the migration of CRC cells by further activating TNS4 and EGFR[92]. For nonhistone arginine methylation, PRMT1 induces asymmetric demethylation of the R251 site of NONO, and compared to NONO WT cells, NONO R251K mutant-expressing CRC cells show reduced migration and invasion. Pharmacological inhibition of PRMT1 significantly reduces the ADMA level of NONO and abrogates the malignant phenotype associated with NONO R251 ADMA in both KRAS WT and KRAS mutant CRC cells[93].

PRMT5 modulates CRC cell migration ability through multiple pathways, including NF-kB/p65 signaling and EGFR/Akt/GSK3β signaling[94, 95]. However, the modification sites mediated by PRMT5 are not discussed in these articles. PRMT5 also has a synergistic effect with different kinds of modifications to promote CRC metastasis. SIRT7-mediated K3 and K243 deacetylation of WDR77 reduces WDR77 interaction with PRMT5 and further affects the transmethylase activity of the WDR77/PRMT5 complex, resulting in a reduction in H4R3me2 modification, which is related to the migration ability of CRC cells[96]. Although PRMT5 has been reported to methylate H3R8, H3R2, and H4R3 to facilitate transcriptional activation or regression in cancer[97], there are few studies related to the exact site methylated by PRMT5 in the process of CRC metastasis. Therefore, further studies are urgently needed.

### Lysine-specific demethylase 1 (LSD1)

LSD1 was the first-discovered histone demethylase, and it mainly demethylates mono- or dimethylated H3K4 and K3K9[98]. In addition, it has also been proven to demethylate some nonhistone functional proteins, such as p53, E2F1, and DNMT1[98]. The catalytic activity of LSD1 resides in the AO domain and is dependent on its cofactor flavin-adenine dinucleotide (FAD)[99].

Several studies have shown that LSD1 is aberrantly expressed in multiple cancer types and has great significance in the process of cancer metastasis[100–102]. In CRC, the role of the LSD1 is controversial. Some studies reported that a higher level of LSD1 predicted a better outcome, and a lack of LSD1 significantly correlated with lymph node metastasis or advanced tumor stage[103, 104]. However, many studies have shown that LSD1 plays a prometastatic role in CRC. LSD1 induces demethylation of H3K4me2 at the CDH1 promoter, downregulates CDH1 expression, and consequently accelerates the EMT process[100]. TSPAN8 is a metastasis-promoting tetraspanin that coordinates with CD151 to promote cancer metastasis by recruiting MMP9 and MMP13[105]. LSD1 upregulates TSPAN8 expression by reducing H3K9me2 enrichment at the TSPAN8 promoter and further promotes the EMT process[106]. In addition, it has been reported that LSD1 interacts with Slug (EMT-related transcription factor) and represses the promoter activity of E-cadherin to promote invasion and migration of CRC cells, but whether this function depends on LSD1-mediated histone demethylation was not discussed in this paper[107]. LSD1 not only increases histone demethylation but also enhances the demethylation of nonhistone proteins to promote CRC metastasis. RIOK1 is an atypical serine/threonine kinase. RIOK1 increases the invasion and migration of CRC cells and promotes lung metastasis in vivo[108]. LSD1 demethylates RIOK1 to reverse SETD7-mediated RIOK1 methylation-dependent degradation, thus increasing its stability[108].

### Jumonji domain-containing proteins (JMJD)

JMJD proteins are a family of histone demethylases containing the JMJC catalytic domain. Most of these family members are identified to mainly demethylate H3K4, H3K9, H3K27, H3K36 and H4K20, and a small group of JMJD proteins can also demethylate H3R2 and H4R3[98]. Several studies have shown that JMJD proteins affect the development of many cancer types[98, 109]. In CRC, the JMJD-driven mechanisms of metastasis have also been described in many articles.

A previous study reported that the levels of KDM4C mRNA (encoding JMJD2C protein) were significantly correlated with TNM stage, distant metastasis, OS and tumor recurrence in CRC[110]. In this study, JMJD2C increased the migration rate of CRC cells in vitro and promoted lung metastasis in vivo. JMJD2C is mainly located in the nuclei of CRC cell lines and decreases the levels of H3K9me3 and H3K36me3 on the MALAT1 promoter to enhance the transcriptional level of MALAT1, which promotes tumor growth and metastasis in CRC[110]. JMJD2D, another member of the JMJD family, is significantly upregulated in human colorectal tumor tissues versus control normal tissues and correlates with the level of proliferating cell nuclear antigen[111]. JMJD2D promotes CRC metastasis via multiple pathways, such as the Wnt/β-catenin signaling pathway, hedgehog signaling pathway and glycolysis[111–113]. In these pathways, JMJD2D demethylates the methyl groups of H3K9me3 at the promoters of β-catenin and its’ target genes (Myc, MMP9), Gli2, mTOR, HIF1 β, and PGK1 to increase their transcription and exert its prometastatic function[111–113]. JMJD1A was discovered to be an independent prognostic marker of CRC, and its expression levels were also significantly associated with lymph node metastasis, lymphatic invasion, venous invasion, and the depth of tumor invasion[114]. These signatures prompted researchers to explore the mechanism by which JMJD1A promotes CRC metastasis. They found that the demethylase activity of JMJD1A was required for CRC metastasis that it decreased H3K9me2 levels at the promoters of β-catenin, c-myc and MMP9 genes to activate Wnt/β-catenin signaling [115].

**Table 1:** Enzymes related to posttranslational modification and their mechanisms in CRC metastasis

	Upstream regulator	Epigenetic enzymes	Targets	Biological outcomes	References
Acetylation	LncRNA SATB2-AS1	p300	Histone H3K27 and H3K9	Upregulates the expression of SATB2	[16]
	CREPT	p300	Histone H3K27 and H4	Binds to the c-myc promoter to directly control its transcription efficacy	[18]
	CREPT	p300	β-catenin	Induces the acetylation of β-catenin and increases its stability	[18]
	ArhGAP30	p300	K382 of P53	Upregulates p53 activity	[20]
	TPO	p300	K802 of LRP6	Triggers the tyrosine phosphorylation of LRP6 to activate Wnt signaling	[22]
	-	CBP	K358 of DOT1L	Protects DOT1L from degradation	[19]
	-	PCAF	CXCL12	Upregulates the expression of CXCL12	[36]
	-	PCAF	K19, K49 of β-catenin	Increases the transcriptional activity and nuclear accumulation of β-catenin	[38]
Deacetylation	-	HDAC3	H3	Increases the Ac-H3 level on the B7x promoter and promotes the interaction of C/EBP-α with the promoter region of the B7x gene to upregulate the expression of B7x	[44]
	-	HDAC2 and HDAC3	-	Correlates with the MDSC function and the expression of recruitment-associated genes ARG1, CCR2, and ITGAL	[45]
	-	SIRT1	Fra-1 (acetylation status was not discussed)	Regulates the expression of Fra-1	[49]
	-	SIRT1	miR-1185-1	Decreases H3K9ac enrichment on the promoter of miR-1185-1	[52]
	miR-199b	SIRT1	CREB	Upregulates transcriptional activity of CREB	[53]
	-	SIRT6	snail, H3K9	Directly interacts with snail and works as a reader; suppresses TET1 transcription by modulating H3K9 deacetylation	[50]
	-	SIRT2	K224 of IDH1	Inhibits the enzymatic activity of IDH1	[59]
	-	SIRT4	miR-200c	Affects the expression fo E-cadherin	[60]
methylation	TGF- β-MTA1-SOX4 signaling	EZH2	-	Regulates the expression of EMT-associated factors such as E-cadherin, ZO-1, snail, slug	[68]
	Erk/Akt signaling	EZH2	H3K27me3	Mediates the transcription repression of ITGα2 and E-cadherin	[69]
	LncRNA SNHG14/LncRNA MALAT1	EZH2	H3K27me3	Impairs EPHA7/E-cadherin expression through regulating H3K27me3 on EPHA7/E-cadherin promoter	[71, 74]
	miR-101	EZH2	H3K27me3	Recruits H3K27me3 to miR-101 promoter to further repress the expression of miR-101	[76]
	-	SUV39H1	H3K9me3	Not discussed	[89]
	-	SUV39H2	H3K9me3	Directly binds to the SLIT1 promoter and catalyzes H3K9me3 to suppress SLIT1 expression	[81]
	-	PRMT1	H4R3me2	Recruits SMARCA4 to further activate TNS4 and EGFR transcription	[92]
	-	PRMT1	R251 of NONO	Does not affect NONO expression but does affect its oncogenic function	[93]
	-	PRMT5	-	Activates EGFR/Akt/GSK3β signaling and NF-kB/p65 signaling	[94, 95]
	SIRT7	PRMT5	H4R3me2	Not discussed	[96]
demethylation	-	LSD1	H3K4m2	Downregulates CDH1 expression	[100]
	-	LSD1	H3K9me2	Upregulates TSPAN8 expression	[106]
	-	LSD1	Slug	Represses E-cadherin promoter activity in a Slug-dependent manner	[107]
	-	LSD1	RIOK1	Reverses SETD7-mediated RIOK1 methylation-dependent degradation, thus increasing its stability	[108]
	-	JMJD2C	H3K9me3 and H3K36me3	Increases the transcript level of MALAT1	[110]
	-	JMJD2D	H3K9me3	Promotes the transcription of β-catenin target genes, Gli2, mTOR, HIF1 β, and PGK1	[111–113]
	-	JMJD1A	H3K9me2	Decreases the H3K9me2 level at the promoters of the β-catenin, c-myc and MMP9 genes to activate Wnt/β-catenin signaling	[115]

## DNA modification

### DNA methyltransferases (DNMTs)

DNA methylation is an epigenetic process in which a methyl group transfers onto the C5 position of the cytosine to form 5-methylcytosine (5mC) under the action of DNMTs. DNA methylation mainly occurs on CpG islands. In mammalian genomes, it has been reported that approximately 60 − 70% of CpG islands can be methylated[116]. The function of DNA methylation is closely related to maintaining the stability of genetic information, transcriptional inhibition and activation, X chromosome inactivation, reprogramming mammalian development and some diseases, such as neurological disorders and immunodeficiency[117]. Aberrant DNA methylation frequently occurs in cancer and is associated with tumor suppressor gene silencing and prevents their activation.

DNMT1, DNMT3A and DNMT3B are the most frequently studied DNMTs in CRC, and they contribute to CRC metastasis through various mechanisms (Table 2). To date, several studies have focused on the interaction between noncoding RNA and DNA methylation in the development of CRC. The human 14q32 locus encodes metastasis-suppressive miRNAs that suppress the adhesion, invasion, and migration properties of tumor cells and metastatic colonization of distant sites[118]. Oshima and his coworkers found that 14q32 locus-encoded miRNAs were overexpressed in a DNMT1/DNMT3B−/− DKO cell model and that the hypomethylation of MEG3-DMR, which acts as a cis-regulatory element for 14q32 miRNA expression, exhibited constitutive expression of 14q32 miRNAs[119]. Pharmacologic inhibition of DNA methylation by 5-Aza-dC, an inhibitor of DNA methyltransferases, induces 14q32 miRNA expression and restricts CRC liver metastasis[119]. In addition, DNMT1 and DNMT3B can be targeted by some miRNAs, such as miR-342, miR-124, and miR-506, thereby reducing the global DNA methylation level to restore the expression of tumor suppressive genes, such as E-cadherin, MGMT, P16, ADAM23, Hint1, RASSF1A, and RECK, thus further inhibiting the metastatic potential of CRC[120, 121]. Some cytokines, such as interleukins and chemokines, have also been reported to be involved in CRC metastasis. It has been reported that IL-23 selectively promotes the migration and invasion ability of SW620 cells compared with SW480, HT29, and HCT116 cells[122]. Socs3, an inhibitor of IL-23/stat signaling, was found to be differentially methylated in these cell lines, and this effect was DNMT1 dependent[122]. Wendt el al[123] found that CXCL12 was silenced by DNA hypermethylation in primary colorectal carcinomas as well as colorectal carcinoma-derived cell lines. Double knockout of DNMT1 and DNMT3b restored CXCL12 expression. Finally, the authors confirmed that stable expression of CXCL12 in CRC cell lines can significantly reduce metastatic tumor formation. These results further indicated the importance of DNA methylation in the process of CRC metastasis.

### Ten-eleven translocation (TET) family

TET family contains TET1, TET2 and TET3 proteins, which catalyze the successive oxidation of 5mC to 5-hydroxymethylcytosine (5hmC), 5-formylcytosine (5fC), and 5-carboxylcytosine (5caC)[124]. TET proteins have been widely studied in hematological malignancies. Somatic alteration of TET2, TET1/TET2 deficiency (TET1/2 double knockout), and TET2/TET3 disruption (TET2/3 double knockout) can lead to a wide range of myeloid and lymphoid malignancies, late-onset B-cell lymphoma, and rapid and fully penetrant myeloid leukemia[124].

In contrast to the frequent mutation of TET proteins in blood cancers, TET proteins are always downregulated in many cancer types, such as melanoma[125], glioblastoma[126], breast cancer[127] and prostate cancer[128]. In CRC, loss of TET3 expression can coexist with TET3 frameshift mutation, which may be related to the development of CRC with high microsatellite instability (MSI-H)[129]. In addition, TET1 and TET2 are downregulated in BRAFV600E-mutated colon cancers. Overexpression of BRAFV600E in BRAF wild-type CRC cell lines can significantly repress TET1/TET2 expression, which leads to the hypermethylation of CIMP genes to promote the development of CpG island methylator phenotype colon cancer (CIMP-CC)[130]. Ma et al[131] found that the downregulation of TET1 inhibited the migration of CRC cells; they further found that TET1 regulates hypoxia-responsive genes such as VEGF, Glut1, and EPO by mediating the binding of HIF-1α to hypoxia-response element (HREs) of these genes by changing their CpG methylation levels (Table 2).

**Table 2:** Enzymes related to DNA modification and their functions

	Methylase/ demethylase	Downstream targets	Functions	References
DNA methylation	DNMT1	ADAM23, Hint1, RASSF1A, and RECK	Affects the proliferation, G0/G1 cell cycle arrest and invasion of CRC cells	[121]
		Socs3	Affects the migration and invasion of SW620 cells induced by IL-23	[122]
	DNMT1 and DNMT3b	MEG3-DMR	DNMT1/DNMT3b double knockout increases 14q32 miRNAs expression and inhibits CRC liver metastasis	[119]
		E-cadherin, MGMT and P16	Affects the proliferation, migrative and invasive ability and chemosensitivity of CRC cells	[120]
		CXCL12	Ablation of both DNMT1 and DNMT3b restores CXCL12 expression and further reduces metastatic tumor formation in mice	[123]
DNA demethylation	TET1	VEGF, Glut1, EPO	Mediates the binding of HIF-1α to HREs of these target genes and increases the migration ability of CRC cells	[131]

### N6-methyladenosine (m6A) modification

m^6^A is the most prevalent internal modification in mRNAs and noncoding RNAs (ncRNAs) in higher eukaryotes and is highly conserved in eukaryotes[132]. Several lines of evidence indicate that m^6^A modification is present on lncRNAs, circRNAs[133], and pre-miRNAs[134] and suggest the importance and potential therapeutic value of m^6^A modification. In CRC, it was reported that m^6^A modification accelerates CRC progression by facilitating the glycolytic process of cancer cells[135, 136], inhibiting the immune response of the tumor microenvironment[137], maintaining tumor stem cells and promoting chemoresistance[138–140].

**Fig. 2:**
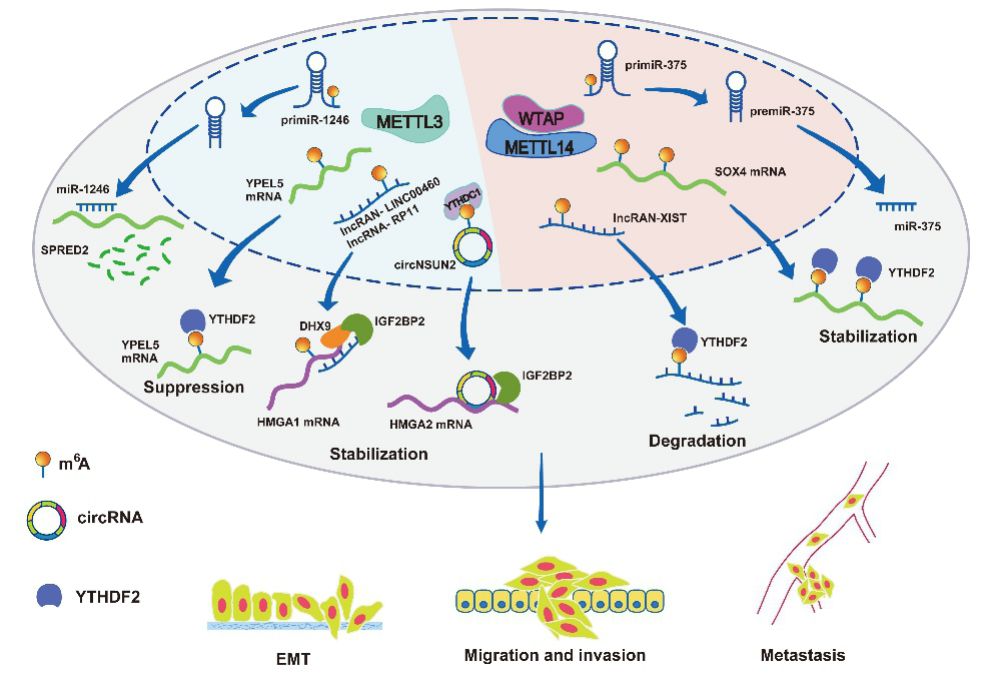
METTL14- and METTL3-mediated m6A modification promotes CRC cell metastasis via various targets and mechanisms

Methyltransferase-like3 (METTL3) and methyltransferase-like 14 (METTL14) are the most widely studied m6A writers in recent years. In CRC, METTL14 is remarkably downregulated in cancerous compared to paired normal samples. Decreased expression of METTL14 is positively correlated with larger tumor size, lymphatic invasion, and remote metastasis and implicates worse RFS[133, 141]. Furthermore, data from The Cancer Genome Atlas (TCGA) showed that METTL14 was positively correlated with OS and was an independent risk factor[141]. These results suggest that METTL14 is a reliable prognostic marker of CRC patients. METTL14 inhibits CRC metastasis by regulating multiple targets (Fig. 2). SOX4 is a downstream target of METTL14. METTL14 knockdown enhances SOX4 mRNA stability in an m6A-YTHDF2-dependent manner and further promotes the EMT process and PI3K/Akt signaling[142]. In addition, METTL14 inhibits CRC growth and metastasis by downregulating lncRNA XIST. METTL14 forms a complex with WTAP and induces the m6A process to suppress XIST expression through YTHDF2-dependent RNA degradation[141]. METTL14 also inhibits CRC metastasis via the miR-375/SP1 pathway[143]. METTL14-dependent m6A methylation enhances pre-miR-375 binding to DGCR8, thereby promoting the DGCR8-mediated maturation of pre-miR-375, which further targets SP1 to accelerate cell invasion and migration[143].

METTL3 is likely to be upregulated in CRC. High METTL3 expression correlates with lymph node invasion and distant metastasis. METTL3 enhances the metastatic potential of CRC cells by promoting m6A modification on pri-miR-1246 to upregulate the level of mature miR-1246, thereby affecting the function of its target gene SPRED2 and the activity of the MAPK pathway[133, 144]. Zhou et al[145] found that METTL3/YTHDF2-mediated m6A modification suppressed YPEL5 expression, which further regulated the PCNA and CCNB1 levels. LINC00460 enhances the interactions between DHX9 or IGF2BP2 and HMGA1, which leads to the upregulation of HMGA1 and promotes CRC growth and metastasis. Interestingly, this process depends on METTL3-mediated m6A modification of HMGA1 mRNA[146]. Chen and his coworkers identified circNSUN2 for the first time and found that it was positively associated with lymph node metastasis and liver metastasis in a cohort of clinical samples and that knockdown of circNSUN2 in patient-derived xenograft (PDX) CRC models significantly inhibited liver and lung metastasis[133]. YTHDC1, an m6A reader that identifies m6A-methylated circNSUN2, facilitates circNSUN2 export from the nucleus to the cytoplasm in an m6A-dependent manner. Consequently, circNSUN2 interacts with IGF2BP2 to stabilize HMGA2 mRNA. They also found that METTL3 plays an important role in this process, affecting the activity of circNSUN2. Once they mutated the m6A modification site (GAACU) in the circNUSN2-overexpressing construct, the m6A modification level of circNUSN2 was downregulated, and the invasion ability of CRC cells was attenuated. Additionally, METTL3 has also been found to mediate the m6A modification of lncRNA RP11 to trigger the dissemination of CRC cells via upregulation of Zeb1[147]. Taken together, these results reveal the diversity of m6A modification targets and suggest that interfering with the interaction between m6A modification and target RNAs is an important way to inhibit colon cancer metastasis.

METTL14 and METTL3 participate in CRC metastasis by inducing the m6A modification of multiple targets, including mRNAs, pre-miRNAs, miRNAs, lncRNAs, and circRNAs.

## Potential clinical application and targeted therapy for CRC metastasis

Due to the lethality of CRC distant metastasis, some predictive markers with low-cost, rapid, high-accuracy and noninvasive characteristics need to be urgently discovered. Alterations in DNA methylation is a hallmark of CRC. A recent study from the Mayo clinic compared 14 methylated DNA markers (MDMs) in primary and metastatic CRC for feasibility in the detection of distantly recurrent/metastatic CRC in plasma[148]. They found that the levels of 14 selected MDMs (VAV3, CHST2, OPLAH, QKI, PPP2R5C, ARHGEF4, PDGFD, ZNF625, SFMBT2, LRRC4, DOCK10, IKZF1, NDRG4, BMP3) were remarkably similar between paired primary and metastatic CRC samples. Thirteen of the MDMs had high accuracy in detecting primary CRC at all stages, and the sensitivity of the 13 MDMs increased with increasing CRC stage (64% for stage I CRC; 62% and 65% for stage II CRC; 79% and 71% for stage III CRC, and 100% for stage IV CRC)[148]. Moreover, they observed that the trained model of MDMs with or without CEA distinguished patients with recurrent CRC from patients with no radiographic evidence of disease (NED) at the immediate previous follow-up with 80% (44–97%) sensitivity and detected metastatic CRC in patients actively undergoing palliative treatment with 93% (78–99%) sensitivity. The panel of MDMs with or without CEA detected recurrent CRC liver metastases with 100% (86–100%) sensitivity, lung metastases with 89% (52–100%) sensitivity, and peritoneal/nodal metastases with 57% (18–90%) sensitivity. Lesions with RECIST sum > 4 cm and ≤ 4 cm were detected with 100% (81–100%) and 83% (59–96%) sensitivity, respectively. The panel of MDMs with or without CEA detected recurrent rectal cancer with 92% (62–100%) sensitivity, left-sided colon cancer with 95% (75–100%) sensitivity, and right-sided colon cancer with 75% (35–97%) sensitivity[148]. This study indicated that the MDMs model was a highly sensitive, reliable, and stable model for detecting early- and late-stage CRC and a promising model for detecting CRC recurrence and metastasis, which strongly proved the clinical application of MDMs in CRC.

Some clinical trials related to epigenetic modifiers for advanced CRC have been tested to observe their efficacy (Table 3). In this review, we mainly focus on DNMT inhibitors, HDAC inhibitors and EZH2 inhibitors. Azacitidine, decitabine and guadecitabine are inhibitors of DNMT that have shown clinical efficacy in the treatment of hematologic malignancies[149, 150]. In CRC, the combined use of DNMT inhibitors and other therapies can be more effective. In a phase I/II study in refractory CIMP-high metastatic colorectal cancer (mCRC), azacitidine combined with capecitabine and oxaliplatin was well tolerated with high rates of stable disease (SD), although no objective responses were reported[151]. Another phase I study combining guadecitabine (SGI-110) with irinotecan in mCRC patients previously exposed to irinotecan reported that treatment with guadecitabine 45 mg/m2 and irinotecan 125 mg/m2 with growth factor support (GFS) was safe and tolerable in patients with mCRC, and 12/17 evaluable patients had SD as the best response, while one had a partial response (PR)[152]. DNMT inhibitors also showed efficacy when combined with targeted therapy. A phase I/II study of decitabine in combination with panitumumab showed good tolerance and activity (10% patients had PR and 50% had SD) in patients with KRAS wild-type mCRC previously treated with cetuximab[153]. Additionally, some researchers have tried to modulate the CRC immune microenvironment through epigenetic treatment. Unfortunately, the combination of guadecitabine with the GVAX colon vaccine was tolerable but showed no significant immunologic activity in mCRC[154].

HDAC inhibitors have shown clinical efficacy and have been approved for the treatment of hematologic malignancies[155]. Among various HDAC inhibitors, varinostat (a small molecule inhibitor of class I and II HDAC enzymes) has been the most studied in advanced solid tumors and mCRC. A phase I study evaluated the safety and efficacy of varinostat in gastrointestinal (GI) cancer. A total of 16 patients received either vorinostat 300 mg bid for 3 consecutive days followed by 4 rest days per cycle (n = 10) or vorinostat 400 mg qd for 21 consecutive days per cycle (n = 6). They reported that vorinostat 300 mg bid for 3 consecutive days followed by 4 days of rest was better tolerated, and 50% of patients achieved SD[156]. This study indicated that vorinostat may be an active agent in the treatment of GI cancer. However, the efficacy of vorinostat combined with 5-fluorouracil (5-FU)-based chemotherapy seems to be limited[157, 158]. This may be due to 5-FU resistance and dose-limiting toxicity (DLT) in selected patients. Fu and coworkers conducted two phase I studies in solid tumors and found that combined treatment with vorinostat and pazopanib yielded significantly longer PFS and OS in patients with metastatic *TP53* mutant solid tumors, especially in those with metastatic sarcoma or mCRC[159, 160]. This finding supports the combined use of vorinostat and antiangiogenic targeted therapy in TP53-mutant mCRC.

EZH2 inhibitors have shown great therapeutic potency in preclinical models of several cancer types. In recent years, many EZH2 inhibitors have been developed and are undergoing clinical trials[161]. Among them, GSK126 (GSK2816126) and tazemetostat have been clinically tested in advanced CRC. GSK126 is a highly selective, S-adenosyl-methionine-competitive inhibitor of EZH2 that can decrease global H3K27me3 levels and reactivate silenced PRC2 target genes[162]. A phase I study reported that the maximum-tolerated dose (MTD) of GSK126 was 2,400 mg, and modest anticancer activity was observed at tolerable doses in patients with advanced solid tumors (including CRC) or B-cell lymphomas[163]. This finding supports the potential use of EZH2 inhibitors in advanced CRC patients. In January 2020, tazemetostat was approved by the FDA for the treatment of adults and adolescents aged ≥ 16 years with locally advanced or metastatic epithelioid sarcoma not eligible for complete resection[164]. Subsequently, several clinical trials conducted in non-Hodgkin lymphoma showed clinically meaningful and durable responses[165, 166]. However, there are few clinical studies of tazemetostat in solid tumors. A phase I study conducted in B-cell non-Hodgkin lymphoma and advanced solid tumors showed a 38% durable objective response rate in B-cell non-Hodgkin lymphoma but only a 5% durable objective response rate in solid tumors with tazemetostat treatment[167]. This result indicates the limited efficacy of monotherapy with EZH2 inhibitors in advanced solid tumors, and more clinical trials on the combined use of chemotherapy or other targeted therapies with EZH2 inhibitors should be carried out in mCRC.

**Table 3:** Clinical results of epigenetic modifier-targeting treatments in advanced CRC

Tumor type	Epigenetic modifier	Combined regimen	Total evaluable patients	Result	Phase	NCT number
Refractory CIMP high mCRC	Azacitidine	Capecitabine/oxaliplatin	26	SD: 17	I/II	NCT01193517
mCRC	Guadecitabine	Irinotecan	17	PR: 1, SD: 12	I	NCT01896856
KRAS-wild type mCRC	Decitabine	Panitumumab	20	PR: 2, SD: 10	I/II	NCT00879385
mCRC	Guadecitabine	GVAX	15	SD: 2, PD: 13	-	NCT01966289
Gastrointestinal cancer mCRC	Vorinostat	--	16	SD: 8	I	-
mCRC	Vorinostat	5-FU/LV	5	SD: 2, PD: 3	I/II	NCT00336141
mCRC	Vorinostat	5-FU	43	PR: 1, SD: 22	II	NCT00942266
Advanced solid tumors	Vorinostat	Pazopanib	78	PR:4, SD: 11	I	NCT01339871
TP53-mutant advanced solid tumors	Vorinostat	Ixazomib	44	SD: 10, PD: 34	I	NCT02042989
Advanced hematologic and solid tumors (including CRC)	GSK2816126	-	Lymphoma: 17 Solid tumors: 19	Lymphoma PR: 1, SD: 6, PD: 10 Solid tumors SD: 8, PD: 11	I	NCT02082977
Refractory B-cell non-Hodgkin lymphoma and advanced solid tumors (including CRC)	Tazemetostat	-	Lymphoma: 21 Solid tumors: 43	Lymphoma CR: 3, PR:5 Solid tumors CR:1, PR: 1, SD: 3	I	NCT01897571

## Conclusion and outlook

CRC metastasis is an important factor affecting the prognosis of CRC patients, and epigenetic modifications play a pivotal role in this process. In this review, we described the roles of several epigenetic enzymes in CRC and summarized their epigenetic mechanisms in the process of CRC metastasis. However, cancer metastasis is a highly complicated process that cannot simply be explained by the alteration of a single modification. Some studies have pointed out that the epigenome is widely altered in cancer[168, 169], which indicates that multiple epigenetic modifications may dynamically and synergistically affect gene function and promote tumor progression in a time- and space-dependent manner. Unfortunately, there are few studies on the synergistic role of multiple modifications in CRC metastasis. Therefore, more studies are needed to explore these potential synergistic effects to better understand the metastatic process of CRC.

**Fig. 3:**
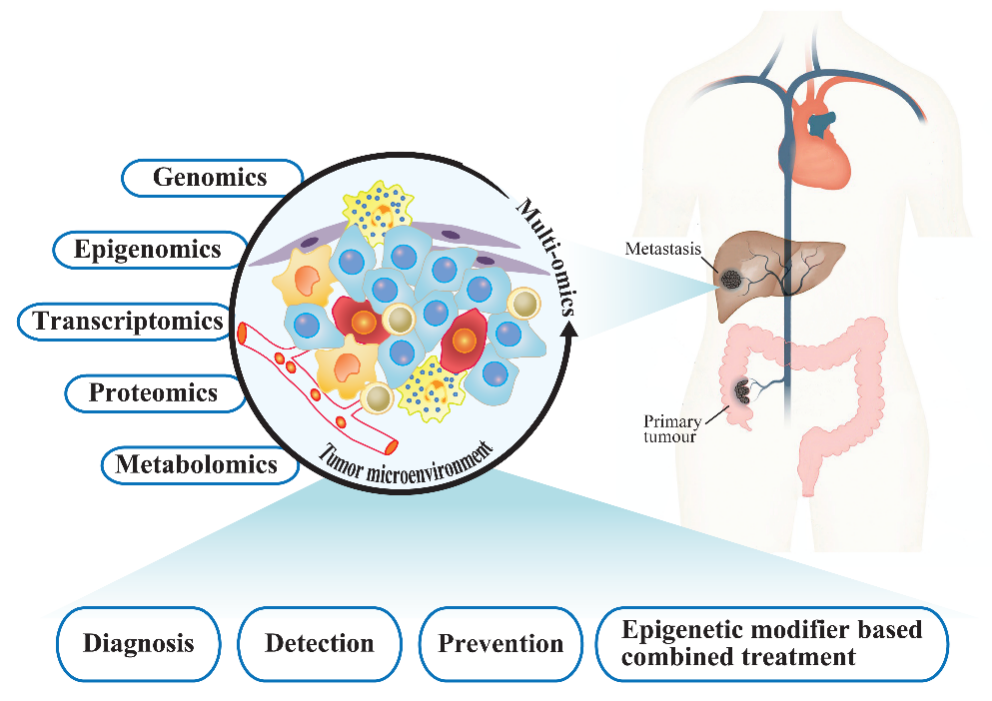
Future direction for the development of epigenetic modifiers based mCRC therapy

In recent years, epigenetic changes have been gradually developed as clinical biomarkers for diagnosis, prognosis and treatment. Epigenetic modifiers have achieved good results in the clinical trials of hematologic malignancies and have received FDA approval for clinical application, suggesting that targeting epigenetic pathways is a promising strategy for cancer treatment. However, in solid tumors, the clinical effect of monotherapy with epigenetic modifiers seems to be limited. This may be due to epigenetic events that cooperate with the driver gene mutations and subsequently result in extensive changes in the tumor microenvironment. The combined use of multiple epigenetic modifiers and/or the combination of epigenetic modifiers with other therapies, such as traditional chemotherapy, targeted therapy and immunotherapy, may improve clinical outcomes. In addition, multiomics analysis of the tumor microenvironment in patients with mCRC, such as epigenomics, genomics, transcriptomics, proteomics and metabolomics analysis, may provide strong scientific evidence for the diagnosis, detection, prevention and epigenetic modifier-based combined treatment of mCRC in the future (Fig. 3).

Multiomics analysis of the tumor microenvironment in patients with mCRC, may provide strong scientific evidence for the diagnosis, detection, prevention and epigenetic modifier-based combined treatment of mCRC.

## Data Availability

No data, models, or code were generated or used during the study.
